# Supplementary material for: Adaptive elastic convolution-based YOLO for peripheral blood smear cell detection
Source: PLoS One. 2026 May 12;21(5):e0347123. doi: 10.1371/journal.pone.0347123 (PMC13166954; doi:10.1371/journal.pone.0347123)
Supplement: S1 Fig — (PDF) [file pone.0347123.s001.pdf]

Supporting Information

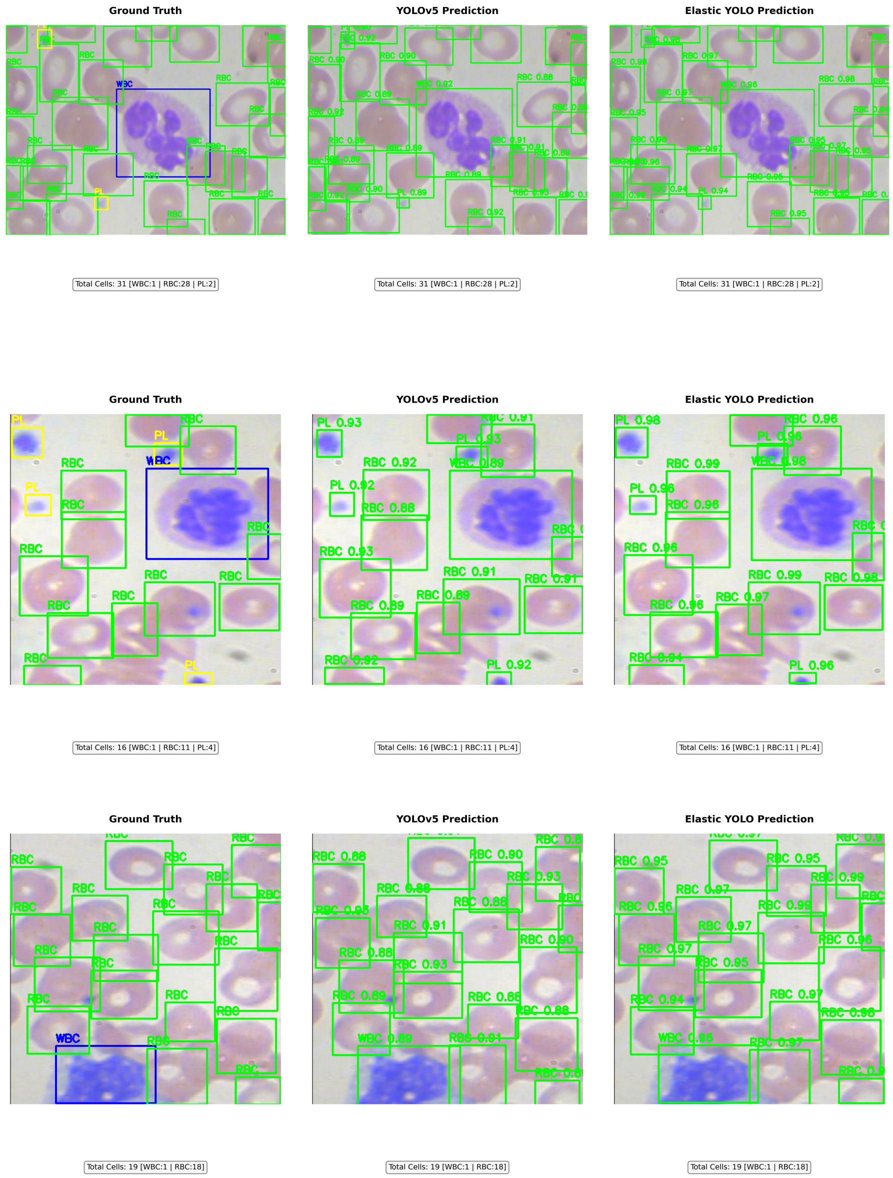

Figure 1. S1 Fig (Part A). Additional qualitative detection examples illustrating Elastic YOLO performance on **normal peripheral blood smear images**. Comparisons between ground truth annotations, YOLOv5 predictions, and Elastic YOLO predictions are shown. Bounding boxes indicate detected cell instances with corresponding class labels and confidence scores. Elastic YOLO demonstrates improved localization consistency under dense cell distributions and overlapping morphologies.

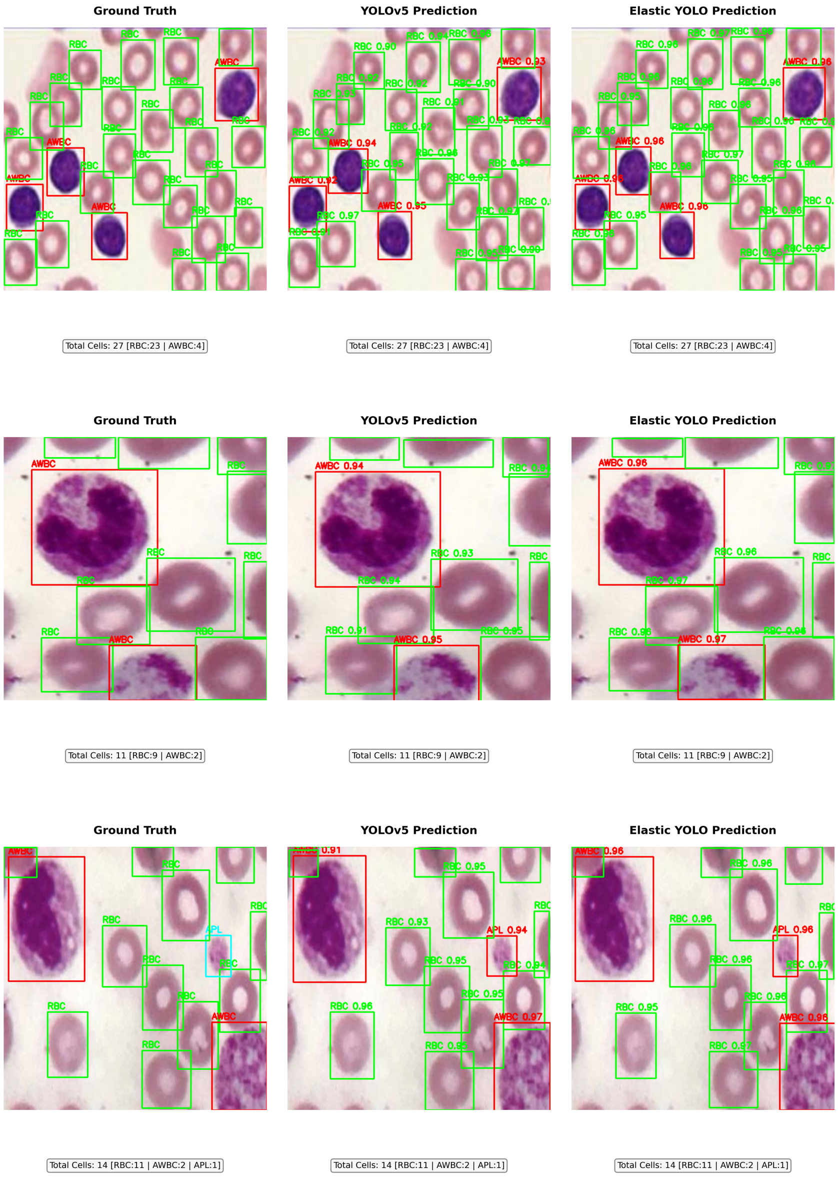

Figure 2. S1 Fig (Part B). Additional qualitative detection examples illustrating Elastic YOLO performance on **abnormal peripheral blood smear images** across diverse staining conditions and pathological variations. Elastic YOLO exhibits enhanced robustness and precise localization of abnormal cell morphologies compared to the baseline YOLOv5 model.
