# Supplementary material for: Adaptive elastic convolution-based YOLO for peripheral blood smear cell detection
Source: PLoS One. 2026 May 12;21(5):e0347123. doi: 10.1371/journal.pone.0347123 (PMC13166954; doi:10.1371/journal.pone.0347123)
Supplement: S1 Table — (PDF) [file pone.0347123.s002.pdf]

Supporting Information

Table 1. S1 Table. Extended per-class precision, recall, and IoU values across multiple IoU thresholds

Class	P@0.5 (%)	R@0.5 (%)	IoU@0.5 (%)	IoU@0.75 (%)	IoU@0.5:0.95 (%)
RBC (Normal)	96.1	94.7	90.4	88.6	85.9
RBC (Abnormal)	93.9	91.7	93.9	91.2	88.1
WBC (Normal)	97.0	95.2	91.8	89.9	86.7
WBC (Abnormal)	94.5	92.3	88.2	85.6	82.4
PL (Normal)	95.0	93.2	89.6	87.1	83.8
PL (Abnormal)	92.4	90.1	86.0	83.2	79.6
**Mean**	**94.8**	**92.9**	**90.0**	**87.6**	**84.4**

Table notes: Precision (P) and Recall (R) are reported at an Intersection over Union (IoU) threshold of 0.5. IoU@0.75 reflects stricter localization accuracy, while IoU@0.5:0.95 represents COCO-style averaged localization performance across multiple thresholds. Normal cell classes exhibit higher spatial agreement, whereas abnormal classes show slightly reduced IoU due to morphological irregularities, overlap, and staining variability inherent in pathological samples.
