# Supplementary material for: Adaptive elastic convolution-based YOLO for peripheral blood smear cell detection
Source: PLoS One. 2026 May 12;21(5):e0347123. doi: 10.1371/journal.pone.0347123 (PMC13166954; doi:10.1371/journal.pone.0347123)
Supplement: Attachment — Submitted filename: Comments to Authors.pdf [file pone.0347123.s003.pdf]

Comments to the Authors: PONE-D-25-67456

This manuscript describes an adaptive Elastic YOLO framework for automated detection and classification of normal and abnormal peripheral blood smear cells. The study is technically rigorous, includes appropriate benchmarking and ablation analyses, and the conclusions are generally supported by the data. The suggested revisions will improve clarity, transparency, and interpretability. **Minor Revision is required.**

Major Comments

1. Clinical Interpretation of Findings: Please moderate statements implying clinical or telemedicine uses, as prospective or multi-center validation and comparison with expert hematologists are not presented.
2. Dataset Composition and Generalizability: Include a brief discussion on potential dataset bias, domain shift, and generalizability across laboratories, staining protocols, and imaging conditions.
3. Use of Synthetic Data in Training: Provide justification for inclusion of GAN-generated images during training and acknowledge potential limitations.
4. Reproducibility and Data Availability: Clarify whether trained model weights and inference code can be made publicly available in line with PLOS ONE's open-science principles.

Minor Comments

a) Streamline the manuscript to reduce redundancy in methodology and results sections.
b) Consider moving some figures and tables to supplementary materials.
c) Correct minor grammatical and typographical errors.
d) Ensure consistent terminology for abnormal or infected cell classes.
