# Supplementary material for: Adaptive elastic convolution-based YOLO for peripheral blood smear cell detection
Source: PLoS One. 2026 May 12;21(5):e0347123. doi: 10.1371/journal.pone.0347123 (PMC13166954; doi:10.1371/journal.pone.0347123)
Supplement: Attachment — Submitted filename: Comments to the Editor.pdf [file pone.0347123.s004.pdf]

Confidential Comments to the Editor: PONE-D-25-67456

This manuscript presents a technically sound and comprehensive deep-learning framework (Elastic YOLO) for multiclass detection of peripheral blood smear cells. The methodology is clearly described, results are supported by extensive quantitative analyses, and comparisons with established YOLO variants are appropriate. Overall, the study meets the criteria for technical validity and reproducibility.

The remaining issues relate primarily to clarity, moderation of claims, and transparency rather than fundamental methodological concerns. Statements suggesting near-term clinical use should be moderated to align with the available data, and dataset heterogeneity and reproducibility aspects should be further clarified. These points can be addressed through minor revisions. I therefore recommend **Minor Revision.**
