# Supplementary material for: Adaptive elastic convolution-based YOLO for peripheral blood smear cell detection
Source: PLoS One. 2026 May 12;21(5):e0347123. doi: 10.1371/journal.pone.0347123 (PMC13166954; doi:10.1371/journal.pone.0347123)
Supplement: Attachment — Submitted filename: Response to Reviewers.pdf [file pone.0347123.s007.pdf]

Manuscript Number: PONE-D-25-67456

Original Article Title: "Adaptive Elastic Convolution-Based YOLO for Peripheral Blood Smear Cell Detection."

To: PLOS ONE Editor

Re: Response to Reviewers and Resubmission

Dear Editor,

We sincerely thank you and the reviewers for the careful evaluation of our manuscript and for the constructive comments provided during the review process. We appreciate the opportunity to revise and resubmit our work.

We have carefully addressed all reviewer comments and substantially revised the manuscript to improve its clarity, scientific rigor, clinical framing, and overall presentation. In particular, we have moderated clinical claims, strengthened methodological transparency, clarified dataset composition and annotation scope, and improved accessibility for a broad interdisciplinary readership, as suggested by the reviewers.

We are uploading (a) our point-by-point response to the comments (below) ("Response to Reviewers"), (b) an updated manuscript with yellow highlighting indicating changes (as "Revised Manuscript with Track Changes"), and (c) a clean updated manuscript without highlights ("Manuscript"*).*

We believe that the revised manuscript now adequately addresses all concerns raised by the reviewers and is well aligned with the scope and standards of *PLOS ONE*. We hope that the revisions meet the reviewers' expectations and improve the overall quality and clarity of the work.

Thank you very much for your time and consideration of our resubmission. We look forward to your feedback.

Sincerely,

Rajakumar K,
School of Computer Science and Engineering,
V. I. T – University, Vellore, 632014,
Tamil Nadu, India.
(Email Id: rajakumar.krishnan@vit.ac.in)

Academic Editor, Concern # 1 (Please ensure that your manuscript meets PLOS ONE's style requirements, including those for file naming. The PLOS ONE style templates can be found at the provided links.):

Author response: Thank you for highlighting the importance of strict adherence to PLOS ONE formatting and style guidelines.

Author action: We have carefully revised the manuscript to fully comply with PLOS ONE style requirements, including document structure, section formatting, figure and table placement, equation formatting, and file naming conventions, in accordance with the official PLOS ONE templates for the main body and title/authors/affiliations.

Academic Editor, Concern # 2 (Please provide additional details regarding participant consent, including whether consent was informed, the type of consent obtained, and whether data were anonymized or consent was waived by the ethics committee.):

Author response: We appreciate the request for clarification regarding ethical considerations and consent procedures.

Author action: We have revised the Ethics Statement to explicitly clarify that this study did not involve prospective recruitment of human participants, clinical interventions, or direct patient interaction. All peripheral blood smear images were obtained from publicly available datasets and archived laboratory samples that were fully anonymized before access. As the data were retrospective and anonymized, the requirement for informed consent was waived in accordance with institutional and international ethical guidelines. No minors were involved in this study. ***(Please refer to line nos. 619-628.)***

Academic Editor, Concern # 3 (PLOS ONE has specific guidelines on code sharing for submissions in which author-generated code underpins the findings in the manuscript.):

Author response: Thank you for drawing attention to the code-sharing policy and reproducibility requirements.

Author action: We have revised the Data Availability section to clarify that the study was conducted using publicly available deep learning frameworks and open-source YOLO implementations. Model training and evaluation were performed through configuration-based adaptations of existing open-source code rather than the development of standalone proprietary software. All architectural details, hyperparameters, preprocessing steps, and evaluation protocols required to reproduce the results are fully described within the manuscript, enabling independent replication without restrictions. ***(Please refer to line nos. 606-618.)***

Academic Editor, Concern # 4 (You indicated that data will be made available upon acceptance. Please revise the statement to clarify data availability and compliance with PLOS ONE's open data policy.):

Author response: We thank the editor for this important clarification regarding data availability expectations.

Author action: We have revised the Data Availability Statement to clearly specify that all datasets used in this study are publicly available (TXL-PBC dataset and ASH Image Bank) or derived from fully anonymized archived laboratory samples used in compliance with licensing and ethical guidelines. No access restrictions

apply to the data required to reproduce the reported results. This revision ensures full compliance with PLOS ONE's open data policy. ***(Please refer to line nos. 606-611.)***

Academic Editor, Concern # 5 (Please amend your manuscript to include a reference list placed at the end of the manuscript and numbered in the order of appearance.):

Author response: Thank you for pointing out this essential formatting requirement.

Author action: We have ensured that a complete reference list is included at the end of the manuscript, with references numbered sequentially in the order of first appearance in the text and formatted according to PLOS ONE reference style guidelines.

Academic Editor, Concern # 6 (We note that a table has been included that is not referenced in the text. Please ensure that Table 3 is cited.):

Author response: We appreciate the careful review of internal cross-referencing.

Author action: We have revised the manuscript to explicitly cite Table 3 at the appropriate location in the text, ensuring consistency between the narrative discussion. ***(Please refer to line nos. 373.)***

Academic Editor, Concern # 7 (Please include captions for your Supporting Information files at the end of the manuscript and update in-text citations accordingly.):

Author response: Thank you for this observation regarding Supporting Information documentation.

Author action: We have added appropriate captions for all Supporting Information files at the end of the manuscript and updated all corresponding in-text citations to ensure consistency with PLOS ONE Supporting Information guidelines. ***(Please refer to line nos. 597-601.)***

Academic Editor, Concern # 8 (We are unable to open the Supporting Information files [plos2025.bst, plos_bibtex_sample.bib].):

Author response: We regret the inconvenience caused by the inaccessible Supporting Information files.

Author action: We have revised and re-uploaded the Supporting Information files to ensure that they are accessible, properly formatted, and compatible with the submission system.

Academic Editor, Concern # 9 (If reviewer comments include a recommendation to cite specific previously published works, please evaluate whether they are relevant.):

Author response: Thank you for the guidance regarding citation practices.

Author action: We carefully reviewed all suggested references mentioned in the reviewer comments and incorporated those that were directly relevant to the scope, methodology, and discussion of this study. References deemed not directly relevant were not included, in accordance with editorial guidance.

Reviewer#1, Concern # 1 (Elastic YOLO should aim for lightweight, high-precision deployment. The system achieves a throughput of 78 FPS, 30.1 GFLOPs, and 12.4M parameters on a NVIDIA RTX 4090 GPU, occupying approximately 110 MB of storage, demonstrating a favorable balance for real-time analyzers and telemedicine platforms.):

Author response: Thank you for acknowledging the lightweight and high-precision design goals of Elastic YOLO and for recognizing the achieved balance between computational efficiency and detection performance.

Author action: We have further emphasized the lightweight deployment intent of Elastic YOLO in the manuscript by explicitly contextualizing the reported throughput, FLOPs, parameter count, and model footprint with respect to real-time hematology analyzers and telemedicine-based diagnostic workflows. The discussion now clearly highlights how the achieved efficiency–accuracy trade-off supports practical deployment scenarios. ***(Please refer to line nos. 359, 366-371, 553-561.)***

Reviewer#1, Concern # 2 (The authors acknowledge that current challenges include soft boundaries and overlapping cells. Future work will focus on Explainable AI (XAI), high-resolution processing, and attention modules to enhance clinical credibility and transparency.):

Author response: We appreciate the reviewer's recognition of the identified limitations and the proposed future research directions.

Author action: We have refined the Discussion and Future Directions sections to clearly articulate the limitations arising from soft cell boundaries and overlapping cellular clusters, and we have expanded the future work discussion to explicitly include Explainable AI (XAI), high-resolution image processing, and attention-based mechanisms as planned extensions to improve interpretability, robustness, and clinical trustworthiness. ***(Please refer to line nos. 567-572, 590-594.)***

Reviewer#1, Concern # 3 (Absence of an All-Inclusive Framework: Current models often focus on classifying single cells or specific morphological features, rather than providing a comprehensive framework for multi-class blood cell detection. This limits their applicability to the full spectrum of hematological disorders.):

Author response: Thank you for highlighting this important limitation in existing blood cell detection literature.

Author action: We have strengthened the Related Work, Discussion, and Contribution sections to explicitly position Elastic YOLO as a unified, all-inclusive framework capable of simultaneously detecting multiple blood cell types (RBCs, WBCs, and platelets) along with their abnormal variants within a single architecture. The manuscript now clearly contrasts this unified design with prior single-cell or narrowly focused approaches,

emphasizing improved applicability across diverse hematological conditions. ***(Please refer to line nos. 141-155, 174-177, 534-543.)***

Reviewer#1, Concern # 4 (The model occasionally misses small-sized platelets and faint abnormal RBCs, which is attributed to imaging quality and dataset limitations. This indicates that while the model performs well, there are still challenging cases it struggles with.):

Author response: We thank the reviewer for this accurate observation regarding challenging detection scenarios.

Author action: We have expanded the Error Analysis section to explicitly discuss missed detections involving small platelets and faint abnormal RBCs, attributing these cases to inherent imaging quality limitations, low contrast, and dataset imbalance rather than systematic model failure. We also outline potential mitigation strategies, including higher-resolution inputs and attention-based feature refinement, as part of future work. ***(Please refer to line nos. 495-507.)***

Reviewer#1, Concern # 5 (The dataset used in the study is dominated by a large number of annotations for Red Blood Cells (RBCs)):

Author response: We appreciate the reviewer's attention to dataset composition and class distribution.

Author action: We have clarified in the Dataset Description and Statistical Analysis sections that the dominance of RBC annotations reflects real-world peripheral blood smear distributions, where erythrocytes naturally outnumber leukocytes and platelets by several orders of magnitude. The manuscript now explicitly discusses this class imbalance as a clinically realistic characteristic rather than a dataset bias, and highlights how Elastic YOLO maintains robust performance despite this imbalance. ***(Please refer to line nos. 228-233, 250-256, 495-501.)***

Reviewer#2, Concern # 1 (Authors need to clarify the conceptual novelty of Elastic YOLO beyond existing deformable convolution and dynamic scaling approaches, as several architectural elements appear incremental and the manuscript does not clearly articulate what fundamentally new capability is introduced.):

Author response: Thank you for this important observation regarding conceptual novelty and architectural differentiation.

Author action: We have revised the Introduction, Related Work, and Proposed Methodology sections to explicitly clarify the conceptual novelty of Elastic YOLO. The manuscript now emphasizes that the primary contribution lies not in individual components such as deformable convolution or architectural scaling alone, but in their unified integration within a single detection framework. We explicitly describe how Elastic YOLO jointly adapts spatial receptive fields and architectural capacity in response to cellular morphological complexity, enabling dynamic allocation of representational resources rather than relying on fixed, design-time assumptions. This coupling introduces a fundamentally new capability for morphology-aware blood cell detection that goes beyond incremental architectural extensions. ***(Please refer to line nos. 54-60, 70-72, 266-270, 296-299.)***

Reviewer#2, Concern # 2 (Authors need to strengthen the clinical framing by explicitly stating which hematological decisions or diagnostic workflows this model is intended to support, since the current emphasis on detection metrics is not sufficiently connected to clinical relevance.):

Author response: We appreciate the reviewer's suggestion to more clearly connect the technical contributions to real-world clinical workflows.

Author action: We have strengthened the clinical framing in the Discussion section by explicitly positioning Elastic YOLO as a computer-assisted decision support tool intended to augment routine hematology workflows. The revised manuscript now clearly states that the model is designed to support peripheral blood smear review triggered by automated analyzer flags, aiding in rapid localization and triage of abnormal cells such as atypical leukocytes, abnormal erythrocytes, and platelet irregularities. This framing connects detection performance metrics to practical use cases including workload reduction, prioritization of diagnostically relevant regions, and improved consistency in smear review. ***(Please refer to line nos. 14-24, 513-521, 522-533.)***

Reviewer#2, Concern # 3 (Authors need to justify the use of GAN generated abnormal samples more rigorously, including a clear discussion of potential bias and evidence that synthetic data does not artificially inflate performance on rare pathological morphologies.):

Author response: Thank you for highlighting the need for greater transparency regarding the use of GAN-generated samples.

Author action: We have expanded the Dataset, Annotations, and Preprocessing section to rigorously justify the use of GAN-generated abnormal samples. The manuscript now clearly states that synthetic images were used exclusively for training-time augmentation to mitigate class imbalance and were never included in validation or test sets. We explicitly discuss potential risks of bias and performance inflation, describe visual inspection procedures to ensure morphological plausibility, and clarify that all reported quantitative results are computed solely on real, clinically sourced images. This ensures that synthetic data does not artificially inflate performance on rare pathological morphologies. ***(Please refer to line nos. 196-205, 612-614.)***

Reviewer#2, Concern # 4 (Authors need to improve methodological transparency by reporting sensitivity or robustness analyses for key elastic parameters such as depth and width scaling, since current ablation studies show component importance but not stability of design choices.):

Author response: We thank the reviewer for pointing out the importance of robustness analysis for elastic design parameters.

Author action: We have extended the Ablation Analysis section to include a dedicated sensitivity analysis for the elastic depth and width scaling parameters. The revised manuscript now reports detection performance stability across moderate variations of these parameters and demonstrates that performance fluctuations remain within a narrow margin. This analysis confirms that Elastic YOLO does not rely on narrowly tuned architectural configurations and that its design choices are robust and stable across reasonable parameter ranges. ***(Please refer to line nos. 475-480, 269, 270.)***

Reviewer#2, Concern # 5 (Authors need to moderate claims regarding real time and telemedicine readiness by evaluating performance on lower resource hardware and across heterogeneous laboratory settings, as current results rely primarily on high end GPU benchmarks.):

Author response: We appreciate the reviewer's guidance regarding responsible framing of deployment readiness.

Author action: We have revised the Discussion and Conclusion sections to moderate claims related to real-time and telemedicine readiness. The manuscript now clearly acknowledges that current performance evaluations are conducted on high-end GPU hardware and that further validation on embedded, lower-resource platforms and heterogeneous laboratory environments is required prior to clinical deployment. We explicitly frame real-time and telemedicine use as future directions rather than immediate deployment claims, ensuring appropriate scientific caution. ***(Please refer to line nos. 553-557, 590-592, 615-618.)***

Reviewer#3, Concern # 1 (This manuscript presents a technically sound and comprehensive deep-learning framework (Elastic YOLO) for multiclass detection of peripheral blood smear cells. The methodology is clearly described, results are supported by extensive quantitative analyses, and comparisons with established YOLO variants are appropriate. Overall, the study meets the criteria for technical validity and reproducibility.):

Author response: We sincerely thank the reviewer for the positive evaluation of the technical soundness, methodological clarity, and reproducibility of the proposed Elastic YOLO framework.

Author action: No changes were required in response to this comment, as it acknowledges the overall technical validity and rigor of the study.

Reviewer#3, Concern # 2 (The remaining issues relate primarily to clarity, moderation of claims, and transparency rather than fundamental methodological concerns. Statements suggesting near-term clinical use should be moderated to align with the available data, and dataset heterogeneity and reproducibility aspects should be further clarified. These points can be addressed through minor revisions.):

Author response: We thank the reviewer for these constructive suggestions and for identifying areas where clarity and transparency could be further improved.

Author action: We have moderated statements related to near-term clinical deployment and telemedicine readiness to ensure alignment with the current scope of experimental validation. The revised manuscript now explicitly acknowledges that real-time performance was demonstrated on high-end GPU hardware and that further evaluation on lower-resource and heterogeneous clinical environments is required prior to clinical deployment. In addition, we have clarified dataset heterogeneity by explicitly describing the multi-source nature of the data, including differences in staining protocols, acquisition conditions, and laboratory environments, as well as the fully anonymized and reproducible nature of the dataset and experimental pipeline. These revisions improve transparency while maintaining the technical conclusions of the study. ***(Please refer to line nos. 590-592, 553-557, 208-210, 211-233, 612-618.)***

Reviewer#4, Concern # 1 (Define YOLO and EYOLO in the Abstract. I don't know how sophisticated the potential readership for this paper will be, but it would be helpful to do so in the abstract, even though you do define it in the third paragraph of the introduction.):

Author response: We thank the reviewer for this helpful suggestion regarding accessibility and clarity for a broad readership.

Author action: We have revised the Abstract to explicitly define both YOLO (You Only Look Once) and EYOLO (Elastic YOLO) at their first occurrence. This ensures that readers unfamiliar with object detection frameworks can clearly understand the terminology without relying on later sections of the manuscript. ***(Please refer to line nos. Abstract Paras: 4-6.)***

Reviewer#4, Concern # 2 (The introductory statements introduce the importance of manual peripheral smear examination. However, this introduction misses the point that modern laboratory practice determines when a manual review is needed and where automated analyzers are weak.):

Author response: We appreciate the reviewer's detailed clinical perspective on modern hematology laboratory workflows.

Author action: The Introduction has been substantially revised to explicitly describe contemporary laboratory practice, emphasizing that highly automated CBC analyzers serve as the primary screening tools and that manual smear review is triggered by analyzer-generated flags. We now clearly explain where automated analyzers perform well (normal RBCs, WBCs, and platelets) and where they exhibit limitations, thereby correctly positioning Elastic YOLO as a decision-support tool for flagged or ambiguous cases rather than a replacement for automated analyzers. ***(Please refer to line nos. 14-24.)***

Reviewer#4, Concern # 3 (The second paragraph contains an erroneous statement: "RBCs are usually of normal appearance, but during the response to a viral infection, atypical lymphocytes often have a striking resemblance to healthy WBCs."):

Author response: We thank the reviewer for identifying this incorrect statement and for providing a detailed morphological clarification.

Author action: We have corrected the erroneous sentence and revised the surrounding paragraph to accurately describe atypical lymphocyte morphology. The revised text now explicitly states that atypical lymphocytes are morphologically distinct from normal lymphocytes, highlighting differences in size, nuclear contour, cytoplasmic features, and clinical context, consistent with established hematopathology references. ***(Please refer to line nos. 29-34.)***

Reviewer#4, Concern # 4 (Manual microscopy review remains essential in several scenarios…):

Author response: We agree with the reviewer that manual microscopy remains indispensable in specific clinical scenarios and that this context is essential for framing the contribution of the proposed system.

Author action: We have incorporated a dedicated discussion in the Introduction and Discussion sections outlining established indications for manual smear review, including abnormal analyzer flags, extreme cell

counts, suspected hematologic malignancies, and clinically discordant cases. This contextualization clarifies that Elastic YOLO is intended to assist in these high-impact scenarios by improving visual triage and localization rather than replacing expert review. ***(Please refer to line nos. 513-521.)***

Reviewer#4, Concern # 5 (Related Work… it would be helpful to define the following terms for those unfamiliar with them: DL-Based, Darknet Framework, PyTorch, Vision Transformers, Affine Transformations.):

Author response: We appreciate the reviewer's suggestion to improve accessibility for interdisciplinary readers.

Author action: We have added a concise explanatory paragraph in the Related Work section defining deep learning (DL), the Darknet framework, PyTorch, Vision Transformers, and affine transformations. These definitions are intentionally brief and placed to enhance readability without disrupting the technical flow of the section. ***(Please refer to line nos. 94-100.)***

Reviewer#4, Concern # 6 (Dataset, Annotations, and Preprocessing: The complexity of WBC abnormalities is minimized… Has EYOLO improved this detection?):

Author response: We thank the reviewer for highlighting the importance of granularity in abnormal WBC characterization.

Author action: We have expanded the Dataset, Annotations, and Preprocessing section to clarify that abnormal WBCs represent a heterogeneous group of morphologically atypical leukocytes (e.g., reactive lymphocytes, blast-like cells, dysplastic forms). While fine-grained subclassification of basophils, monocytes, or blasts is not explicitly annotated in the current dataset, the study's objective is robust abnormal-versus-normal discrimination under conditions that challenge automated systems. This clarification ensures that claims remain aligned with the available annotations while acknowledging clinically relevant limitations. ***(Please refer to line nos. 211-233.)***

Reviewer#4, Concern # 7 (Dataset Structure: … You state that there are only a few abnormalities in the dataset. Please clarify. Detecting standard cell types is of minimal interest.):

Author response: We appreciate the reviewer's emphasis on clinical relevance over routine cell detection.

Author action: We have clarified that the dominance of normal RBC annotations reflects real-world peripheral blood smear distributions rather than dataset bias. The manuscript now explicitly explains that abnormal cells are fewer in number but are clinically critical, and that Elastic YOLO's value lies in improved localization and discrimination of these rare, diagnostically significant abnormalities within dense smear backgrounds. ***(Please refer to line nos.219-233.)***

Reviewer#4, Concern # 8 (Results and Discussion: It is of greater interest to understand the performance of this method in identifying analytic challenges posed by NRBCs, leukemic blasts, lymphomas, viral atypical lymphocytes…):

Author response: We thank the reviewer for this clinically insightful comment.

Author action: We have expanded the Discussion section to explicitly address how Elastic YOLO may assist in analytically challenging scenarios, including abnormal leukocytes, atypical lymphocytes, and other rare populations that frequently trigger analyzer flags. While explicit subclass-level performance metrics are not available due to dataset limitations, the discussion now clearly articulates the potential role of Elastic YOLO as a visual triage and localization aid in cases where automated systems and manual screening are most challenged. Future work directions have been explicitly stated to address NRBCs, blasts, basophils, and monocytes through expanded datasets and finer-grained annotations. ***(Please refer to line nos. 522-533.)***
