# Supplementary material for: Adaptive elastic convolution-based YOLO for peripheral blood smear cell detection
Source: PLoS One. 2026 May 12;21(5):e0347123. doi: 10.1371/journal.pone.0347123 (PMC13166954; doi:10.1371/journal.pone.0347123)
Supplement: Attachment — Submitted filename: Comments to Authors PONE-D-25-67456_R1.pdf [file pone.0347123.s006.pdf]

Minor Revisions for Manuscript: PONE-D-25-67456_R1

Abstract, and summary

1. Bring main key performance numbers and baselines earlier in the abstract.
2. Reduce overlap between abstract and Author Summary; add one concrete clinical use-case like telemedicine triage.

Introduction

1. Shorten disease lists by grouping conditions and emphasize the core gap like morphology, rare cells, and manual PBS burden.
2. Briefly state specific limitations of prior YOLO/CNN PBS work like overlap, subtle variants, stain/domain shifts, etc.

Data and acronyms clarity

1. Summarize datasets: names, sizes, classes, sources, stain/imaging conditions, and clinician annotation process.
2. Ensure all symbols and acronyms are defined once and used consistently.

Metrics definitions

1. Define mAP, AP, IoU, FPS, FPN, PANet, CIoU, etc., at their first use.
2. Ensure consistent notation for Cpos, Cneg, Mpos, Mneg and clearly explain F1, precision, and recall.

Results, limitations, and style

1. Report per-class AP, highlighting abnormal classes (ARBC, AWBC, APL), plus a confusion matrix or brief misclassification table.
2. Expand limitations like dataset size/diversity, centers, stains, lack of external cohort, focus on image-level metrics, and link future work to observed failure cases.
3. Tighten long sentences, fix minor grammar, and standardize terminology (PBS, EYOLO). Also ensure Data Availability, Ethics, and Competing Interests in the text match the submission forms and that references follow journal style.
