# Supplementary material for: Adaptive elastic convolution-based YOLO for peripheral blood smear cell detection
Source: PLoS One. 2026 May 12;21(5):e0347123. doi: 10.1371/journal.pone.0347123 (PMC13166954; doi:10.1371/journal.pone.0347123)
Supplement: Attachment — Submitted filename: Response_to_Reviewers_auresp_2.pdf [file pone.0347123.s008.pdf]

Manuscript Number: PONE-D-25-67456

Original Article Title: "Adaptive Elastic Convolution-Based YOLO for Peripheral Blood Smear Cell Detection."

To: PLOS ONE Editor

Re: Response to Reviewers and Resubmission

Dear Editor,

We sincerely thank you and the reviewers for the careful evaluation of our manuscript and for the constructive comments provided during the review process. We appreciate the opportunity to revise and resubmit our work.

We have carefully addressed all reviewer comments and revised the manuscript to improve clarity, methodological transparency, and overall presentation. We have also clarified the dataset description, moderated clinical claims, and improved the explanation of the proposed method to make the manuscript clearer for a broader readership.

We are uploading (a) our point-by-point response to the comments (below) ("Response to Reviewers"), (b) an updated manuscript with yellow highlighting indicating changes (as "Revised Manuscript with Track Changes"), and (c) a clean updated manuscript without highlights ("Manuscript"*).*

We hope that the revisions have addressed the reviewers' concerns and improved the quality of the manuscript. Thank you for your time and consideration of our resubmission.

Sincerely,

Rajakumar K,
School of Computer Science and Engineering,
V. I. T – University, Vellore, 632014,
Tamil Nadu, India.
(Email Id: rajakumar.krishnan@vit.ac.in)

Reviewer 3, Concern # 1 (Abstract, and Summary

1. **Bring main key performance numbers and baselines earlier in the abstract.**
2. **Reduce overlap between abstract and Author Summary; add one concrete clinical use-case like telemedicine triage.):**

Author response: We appreciate the reviewer's helpful suggestion to improve the clarity of the abstract and reduce redundancy between the abstract and the Author Summary.

Author action: We revised the abstract to present the key performance results and baseline comparison earlier. We also reduced overlapping information between the abstract and Author Summary and added a specific clinical application related to telemedicine-based triage. ***(Please refer to Abstract and Author Summary on page 1.)***

Reviewer 3, Concern # 2 (Introduction

1. **Shorten disease lists by grouping conditions and emphasize the core gap like morphology, rare cells, and manual PBS burden.**
2. **Briefly state specific limitations of prior YOLO/CNN PBS work, like overlap, subtle variants, stain/domain shifts, etc.):**

Author response: Thank you for drawing attention to the need for a clearer description of the research gap in the introduction.

Author action: We revised the introduction to group disease conditions and focused more clearly on the main challenges of peripheral blood smear analysis, including morphological variation, rare abnormal cells, and the workload of manual examination. We also briefly highlighted limitations of previous YOLO/CNN-based PBS studies, such as overlapping cells, subtle variants, and staining/domain variations. ***(Please refer to line nos. 2–15 and 32–37.)***

Reviewer 3, Concern # 3 (Data and acronyms clarity

1. **Summarize datasets: names, sizes, classes, sources, stain/imaging conditions, and clinician annotation process.**
2. **Ensure all symbols and acronyms are defined once and used consistently.):**

Author response: We thank the reviewer for the suggestion to improve clarity regarding datasets and acronyms.

Author action: We have added a clearer summary of the datasets, including their names, sizes, classes, sources, and annotation process by medical experts. In addition, all symbols and acronyms are now defined at their first appearance and used consistently throughout the manuscript. ***(Please refer to line nos. 177–185, 213–215, 216–232, 233, Table 1, and line 28)***

Reviewer 3, Concern # 4 (Metrics definitions

1. **Define mAP, AP, IoU, FPS, PANet, CIoU, etc., at their first use.**

2. **Ensure consistent notations for Cpos, Cneg, Mpos, Mneg, and clearly explain F1, precision, and recall.):**

Author response: Thank you for pointing out the need for clearer definitions of evaluation metrics and notations.

Author action: We have ensured that key evaluation metrics such as mAP, AP, IoU, FPS, PANet, and CIoU are defined when first introduced in the manuscript. We also clarified the notation used for C_{pos}, C_{neg}, M_{pos}, and M_{neg}, and added explanations for F1-score, precision, and recall in the evaluation metrics section. ***(Please refer to line nos. 265–267, 327–330, 336–342, 358, and 371-373.)***

Reviewer 3, Concern # 5 (Results, limitations, and style

1. **Report per-class AP, highlighting abnormal classes (ARBC, AWBC, APL), plus a confusion matrix or brief misclassification table.**
2. **Expand limitations like dataset size/diversity, centers, stains, lack of external cohort, focus on image-level metrics, and link future work to observed failure cases.**
3. **Tighten long sentences, fix minor grammar, and standardize terminology (PBS, EYOLO). Also, ensure Data Availability, Ethics, and Competing Interests in the text match the submission forms and that references follow journal style.):**

Author response: We appreciate the careful review of the results and discussion sections and the helpful suggestions for improving clarity and completeness.

Author action: We have highlighted the per-class performance results, especially for abnormal classes (ARBC, AWBC, and APL), and added a confusion matrix analysis to better illustrate classification behavior. The limitations section has been expanded to discuss dataset size, staining variations, and generalization issues, and future work directions are linked to observed failure cases. We also revised several sentences for clarity, standardized terminology such as PBS and Elastic YOLO, and verified that the Data Availability, Ethics, and Competing Interests statements follow journal guidelines. ***(Please refer to line nos. 425–428, 520–523, 524–537, and 625–635.)***
